# Anticancer and Antibacterial Activity Studies of Gold(I)-Alkynyl Chromones

**DOI:** 10.3390/molecules201119647

**Published:** 2015-10-30

**Authors:** Paweł Hikisz, Łukasz Szczupak, Aneta Koceva-Chyła, Adam Guśpiel, Luciano Oehninger, Ingo Ott, Bruno Therrien, Jolanta Solecka, Konrad Kowalski

**Affiliations:** 1 Department of Thermobiology, Faculty of Biology and Environmental Protection, University of Łódź, Pomorska 141/143, Łódź PL-90236, Poland; pawelhikisz@gmail.com (P.H.); koceva.aneta@gmail.com (A.K.-C.); 2 Department of Organic Chemistry, Faculty of Chemistry, University of Łódź, Tamka 12, Łódź PL-91403, Poland; lukaszszczupak@interia.pl; 3 Laboratory of Biologically Active Compounds, National Institute of Public Health-National Institute of Hygiene, Chocimska 24, Warsaw PL-00791, Poland; aguspiel@pzh.gov.pl (A.G.); jsolecka@pzh.gov.pl (J.S.); 4 Institute of Medicinal and Pharmaceutical Chemistry, Technische Universität Braunschweig, Beethovenstr. 55, Braunschweig D-38106, Germany; l.oehninger@tu-braunschweig (L.O.); ingo.ott@tu-bs.de (I.O.); 5 Institute of Chemistry, Faculty of Science, University of Neuchatel, Avenue de Bellevaux 51, Neuchatel CH-2000, Switzerland; Bruno.Therrien@unine.ch

**Keywords:** anticancer activity, gold(I) complexes, ferrocene, TrxR, caspases, antibacterial activity

## Abstract

Three gold(I) complexes of alkynyl chromones were synthesized and characterized. The single-crystal X-ray structure analysis of a dinuclear compound and of a flavone derivative exhibit a typical d^10^ gold(I)-alkynyl linear arrangement. All complexes were evaluated as anticancer and antibacterial agents against four human cancer cell lines and four pathogenic bacterial strains. All compounds show antiproliferative activity at lower micromolar range concentrations. Complex **4** showed a broad activity profile, being more active than the reference drug auranofin against HepG2, MCF-7 and CCRF-CEM cancer cells. The cellular uptake into MCF-7 cells of the investigated complexes was measured by atomic absorption spectroscopy (AAS). These measurements showed a positive correlation between an increased cellular gold content and the incubation time of the complexes. Unexpectedly an opposite effect was observed for the most active compound. Biological assays revealed various molecular mechanisms for these compounds, comprising: (i) thioredoxin reductase (TrxR) inhibition, (ii) caspases-9 and -3 activation; (iii) DNA damaging activity and (iv) cell cycle disturbance. The gold(I) complexes were also bactericidal against Gram-positive methicillin-sensitive *Staphylococcus aureus* (MSSA) and methicillin-resistant *S. aureus* (MRSA) bacterial strains, while showing no activity against the Gram-negative *Escherichia coli* bacterial strain.

## 1. Introduction

Platinum-based drugs (e.g., cisplatin, oxaliplatin and carboplatin) are an important class of anticancer agents with proven clinical and commercial value [1,2,3]. Promising anticancer active metal complexes include the ruthenium-based derivatives RAPTA-C [4], NAMI-A [5], KP1019 [6], PIM-1 inhibitors [7], and a family of iron-based ferrocifens [8,9]. Although none of these compounds have been introduced to the market so far, they revealed a hidden potential of metal complexes in cancer therapy. In that respect gold complexes also have a role to play. Gold continues to be a metal of exceptional importance as a decorative material and in the monetary system throughout the entire world. For centuries it has also attracted attention of alchemists and physicians [10]. Thanks to Robert Koch’s observations of the bacteriostatic activity of K[Au(CN)_2_], gold complexes have entered modern medicine [11]. Aurothiomalate, aurothioglucose, aurothiosulfate, aurothiopropanol sulfonate and triethylphosphine gold(I) tetraacetylthioglucose are currently in clinical use modifying antirheumatic drugs. In fact, the application of gold thiolates for the treatment of rheumatoid arthritis dates back to the 1930s of the 20th century [12]. In the 1980s the anticancer properties of auranofin were reported [13,14]. Recently we have witnessed a revival of gold complexes in medicinal chemistry and new results have demonstrated that gold complexes are promising anticancer [15,16,17,18] and antimicrobial [19,20] drug candidates. The majority of biologically tested gold complexes focus on gold ions in the +1 oxidation state and generally bound to thiolates [21,22], to various *N*-heterocyclic carbenes (NHC) [23,24,25,26,27], to diphos-type [28] and alkyne [29,30,31,32,33,34,35] ligands. There is also an increased interest in the development of anticancer gold(III)-NHC complexes [36,37,38]. Mechanistic studies revealed mitochondria and the selenoenzyme thioredoxin reductase (TrxR) as a key cellular and molecular targets for gold complexes [18,27,39]. It has been shown that positively charged gold complexes are able to effectively penetrate to the mitochondria due to the negative membrane potential (Δψm) of this organelle [27,40]. In turn, accumulation of the gold metallodrug in the mitochondria and inhibition of the TrxR enzyme [40,41] has significant metabolic consequences, e.g., imbalance of intracellular redox potential, loss of mitochondrial respiration and an increasing of the reactive oxygen species (ROS) concentration [40]. These alterations ultimately lead to the pro-apoptic proteins signalling induction [27,40,42]. In comparison to the other classes of biologically investigated gold(I) complexes [15,18,19,20,23,24,25,26,27,40,41] gold(I)-alkynyl complexes have been studied to the very limited extent [29,30,31,32,33,34,35]. Moreover, to the best of our knowledge to date there has not been any reported data on the antibacterial activity of gold(I)-alkynyl complexes although some *in vitro* antimalarial activity in two strains of *Plasmodium falciparum* has been published [32].

Chromone and its derivatives are plant metabolites of recognized medicinal importance [43,44]. Recently others [45,46,47,48] and our group [49,50,51,52] have demonstrated that adequately designed metallochromones show anticancer [45,46,47,50,51] and antibacterial [48,49] activity, and that they can also be utilized as luminescent probes in bioimaging [52]. Accordingly, the work reported herein was spurred by these interesting results and by the abovementioned medicinal relevance of gold complexes. Herein we report on the novel gold(I)-alkynyl chromone complexes **4**–**6**. These compounds were biologically examined in respect to anticancer and bactericidal activity. The cellular uptake of compounds **4**, **5** and **6** was investigated by atomic absorption spectroscopy (AAS) while the mechanisms of their anticancer activity were studied by their cytotoxic performance, TrxR inhibition, caspase activation, and genotoxic effects. The Au-Fe bimetallic complex **5** provides us with the possibility to investigate whether the presence of two different metal centres may enhance the biological activity of the system in comparison to the corresponding mononuclear congener.

## 2. Results and Discussion

### 2.1. Synthesis of Complexes **4**–**6**

**Scheme 1:**
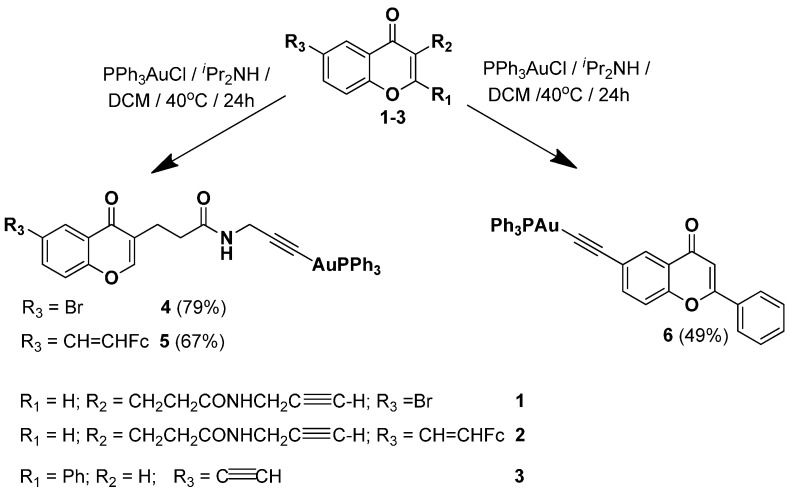
Synthesis of gold(I)-complexes **4**–**6**.

The synthesis of the gold(I)-alkynyl chromones **4**, **5** and gold(I)-alkynyl flavone **6** was carried out according to Scheme 1.

Alkynes **1**, **2** and **3** were obtained according to the literature [50,53]. Their treatment with chloro-(triphenylphosphine)gold(I) complex and diisopropylamine in dichloromethane at 40 °C [54] afforded products **4**, **5** and **6** in 79%, 67% and 49% yield respectively after purification. Gold(I)-alkynes **4** and **6** are colorless solids, while the binuclear complex **5** is an orange solid. The products were characterized by ^1^H-, ^13^C-, ^31^P-NMR, IR spectroscopy, MS and elemental analyses. The analytical data confirm the proposed constitution. The formation of complexes **4**–**6** was evidenced by the disappearance of the signals of the terminal ethynyl protons in the δ 3.15–2.13 ppm range of their ^1^H-NMR spectra. The ^31^P-NMR spectra of **4**–**6** show a single phosphorus signal at *ca.* 42 ppm, similarly to other reported gold(I)-alkynyl complexes [32]. The IR spectra of complexes **4**–**6** show diagnostic ethynyl absorption bands at *ca.* 2119 cm^−1^ [30,32].

### 2.2. X-ray Diffraction Study of Compounds

**Figure 1:**
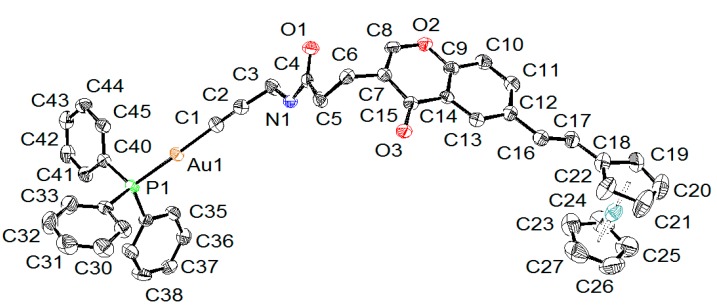
ORTEP drawing of **5** at 50% probability ellipsoids with hydrogen atoms and solvent molecules omitted for clarity.

**Figure 2:**
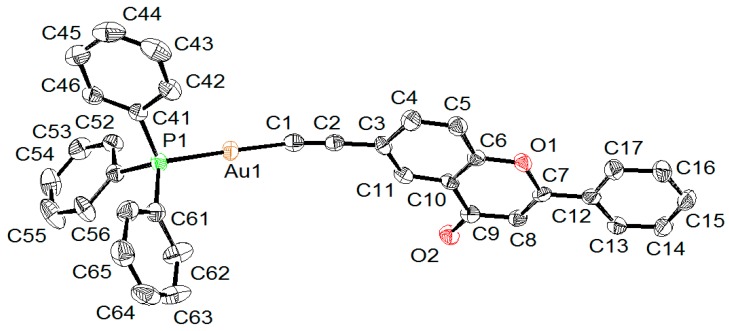
Ortep drawing of **6A** at 50% probability ellipsoids with hydrogen atoms omitted for clarity, only one independent molecule being shown.

The molecular structures of complexes **5** and **6** were determined by single-crystal X-ray structure analyses (Figure 1 and Figure 2). Crystallographic details regarding data collections and structure refinements are summarized in Table S1. In both complexes, the gold atom shows a linear geometry with C-Au-P angles of 178.13(14)° in **5** and 174.00(12)° and 172.46(12)° in **6**, where two independent molecules are observed in the crystal lattice. In both complexes, the Au-C (1.99–2.00 Å) and Au-P (2.27–2.28 Å) distances are considered normal (see Table 1), and overall, the geometrical parameters around the gold atoms are almost identical in both complexes. These geometrical values are consistent with those found in analogous triphenylphosphine organo-gold complexes [29,55].

**Table 1:** Selected bond lengths and angles for **5** and **6** (in **6**, two independent molecules **A** and **B** are found in the crystal lattice).

Compound	5	6A	6B
Interatomic Distances (Å)			
Au-C	1.996(4)	1.989(5)	1.988(4)
Au-P	2.2787(10)	2.2739(12)	2.2720(11)
C≡C	1.205(6)	1.214(6)	1.199(6)
Angles (°)			
P-Au-C	178.13(14)	174.00(12)	172.46(12)
Au-C≡C	176.5(4)	164.3(4)	165.9(4)

In **5**, the ferrocenyl moiety is pointing away from the gold atom, the intramolecular separation between the two metals being 16.999(1) Å. The ferrocenyl unit adopts an eclipsed conformation and is almost coplanar to the chromone moiety: the angle between the plane defined by the functionalized cyclopentadienyl ring (C18–C21) and the chromone plane (C6–C16) being 15.1(3)°. A coplanarity between the chromone moiety and the phenyl functional group is also observed in **6**: the angles between the plane of the phenyl ring and the chromone are respectively 0.54(6)° in **6A** and 1.58(6)° in **6B**. This coplanarity is favored in the solid state due to strong π-stacking interactions between neighboring molecules.

**Figure 3:**
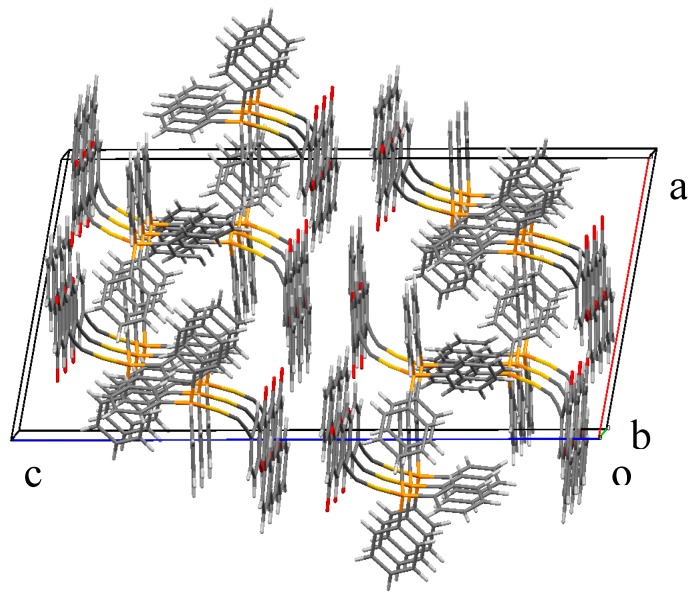
Crystal packing of **6**, showing the planar aromatic layers between the chromone units and the face-to-face arrangement of the triphenylphosphine gold units (o = origin; a, b, c = crystallographic axes).

Indeed, the presence of large planar aromatic units in **6** generates an interesting molecular packing in the crystal (Figure 3). The chromone moieties alligned themselves to form layers, thus forcing the triphenylphosphine gold units to face each other. These large planar aromatic surfaces are only separated by 3.34 Å, providing strong π-π interactions between these layers. On the other hand, no such strong π-stacking interactions are observed in **5** due to the non-planarity of the dinuclear complex and the presence of solvent molecules in the packing of the crystal.

### 2.3. Biological Results

#### 2.3.1. Antiproliferative Effect toward Human Cancer Cells

In the first stage of the study, complexes **4**–**6** and the reference compound auranofin were screened for cytotoxicity against solid (hepatocellular carcinoma HepG2, estrogen-responsive breast cancer cells MCF-7, estrogen-unresponsive breast cancer cells MDA-MB-231) and hematological (T lymphoblast-like CCRF-CEM) human cancer cells. Table 2 lists the IC_50_ concentrations obtained for the entire series of tested compounds.

**Table 2:** IC_50_ concentrations of **4**, **5**, **6** and auranofin against HepG2, MCF-7, MDA-MB-231) and CCRF-CEM cells. The cells were incubated with the compounds for 24 h (cytotoxic effect); results are expressed as means ±SEM of repeated experiments.

Cell Line	IC_50_ (µmol/L)			
	4	5	6	Auranofin
HepG2	10.0 ± 1.3	>120	19.5 ± 1.9	50.0 ± 2.7
MCF-7	5.5 ± 0.7	11.0 ± 0.8	13.0 ± 2.3	13.1 ± 0.9
MDA-MB-231	9.7 ± 2.5	49.7 ± 1,7	10.0 ± 3.4	3.0 ± 0.6
CCRF-CEM	3.5 ± 1.1	3.8 ± 0.9	4.2 ± 0.9	6.0 ± 1.2

Collected in Table 2, the IC_50_ values for the reference auranofin complex are in a low micromolar range, thus indicating the high cytotoxic potency of this drug. The data obtained here show the overall cytotoxicity of conjugates **4**–**6**, comparable to that of auranofin. Noticably, the most active of the series, *i.e.*, complex **4**, showed higher antiproliferative activity than auranofin against HepG2, MCF-7 and CCRF-CEM cells, with the lowest IC_50_ value of 3.5 ± 1.1 for the latter cells. Also, flavone derivative **6** showed higher cytotoxicity against HepG2 and CCRF-CEM cells than the reference drug. The weakest cytotoxic activity was observed for the Au-Fe bimetallic complex **5**. Although it was the less active of the whole series, complex **5** showed an enhancement in activity against the MCF-7 and CCRF-CEM cell lines when compared to that shown by its mononuclear ferrocenyl congener **2** [50]. Gold(I)-alkynyl complexes were also investigated in non-transformed human umbilical vein endothelial cells (HUVEC-ST). Complexes **4**, **5**, and **6** showed IC_50_ values of 2.1 ± 0.9, 3.1 ± 1.1, and 2.9 ± 1.3 μmol/L respectively against HUVEC cells.

The next stage of the study was designed to investigate the molecular mechanisms responsible for the cytotoxicity of gold complexes. These comprise cellular uptake, thioredoxin reductase (TrxR) inhibition, genotoxic effect, caspase activation and cell cycle analysis. Only the most active complexes in the corresponding cell lines were investigated.

#### 2.3.2. Cellular Uptake with High-Resolution Continuum-Source Atomic Absorption Spectroscopy

**Figure 4:**
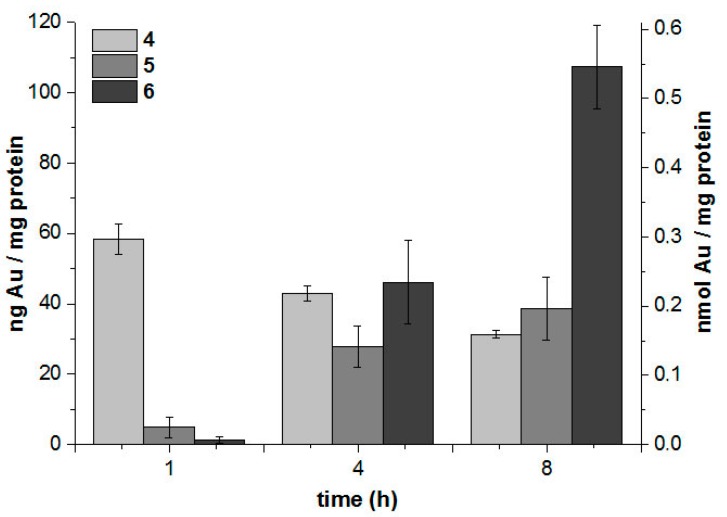
Cellular gold level of MCF-7 cells exposed to 3.0 µM in **4**–**6** determined by HRCS-AAS.

The cellular uptake of complexes **4**–**6** into MCF-7 cells was studied by a method based on gold quantification by high-resolution atomic absorption spectroscopy (HRCS-AAS). For this purpose, the cells were exposed to 3.0 µM of the complexes, and the gold and protein levels of the cells were determined after 1, 4 and 8 h by HRCS-AAS and the Bradford method, respectively. Cellular gold levels are expressed as the amount (ng or nmol, respectively) of gold per mg of cell protein. In general, the obtained values (Figure 4) were within a similar range as compared to previously studied gold-alkynyl species [34].

After a short exposure, complex **4** afforded the highest cellular gold levels, while **5** and **6** showed only marginal uptake. In contrast, after a longer incubation, **5** and **6** afforded increasing values and levels, with **4** decreasing slightly. This effect was most marked for **6**, which afforded the highest cellular accumulation in this study after 8 h of exposure. The observed pattern indicates that the respective compounds undergo different ways of biodistribution and/or metabolisation. However, a correlation with the measured IC_50_ values of cytotoxicity is not apparent, especially in the case of the most active compound **4**.

#### 2.3.3. TrxR Inhibition Assay

**Figure 5:**
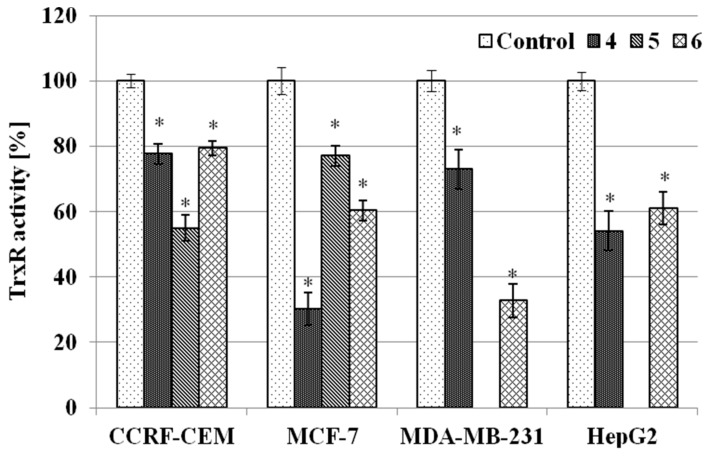
Relative activity of TrxR in human cancer cells treated with complexes **4**, **5** and **6** for 24 h. Enzyme activity was estimated at the end of the incubation period and compared to the untreated control of the corresponding cell line (100%). Each experiment was done at least in triplicate. The results represent means ± SEM of data from 3 to 4 individual experiments, * *p* < 0.05 *vs.* control.

Thioredoxin reductase has been recognised as a key molecular target for organo-gold compounds [18,27,39]. Thus we decided to evaluate our complexes for TrxR inhibition. Data pertaining to the TrxR inhibition assay are shown in Figure 5. Complexes **4**–**6** considerably (*p* < 0.05) inhibited the activity of TrxR. Depending on the cell line and type of complex, a 20%–70% decrease was observed in the enzyme activity. The highest inhibition (70%) was found for complexes **4** and **6** in the MCF-7 and MDA-MB-231 breast cancer cells, respectively. Likewise, complexes **4** and **6** inhibited, to a similar degree (about 20%), TrxR activity in CCRF-CEM cancer cells. The same inhibitory effect was noted for complex **4** in MDA-MB-231 cells and complex **5** in MCF-7 cells. Complex **6** performed similarly in MCF-7 and HepG2 cells (40% inhibition), and the same effect (about 45% inhibition) was found for complex **5** in CCRF-CEM cells and complex **4** in HepG2 cells. The above results show that TrxR is a molecular target for complexes **4**–**6**, which is consistent with findings for other gold derivatives [18,27,39]. A comparison of the cytotoxic effects with TrxR inhibitory effects shows, however, that the cytotoxicity of the gold chromone complexes investigated here is not solely correlated with their effect on TrxR activity. Thus, other mechanisms of action should be considered as well.

#### 2.3.4. DNA Comet Assay

**Figure 6:**
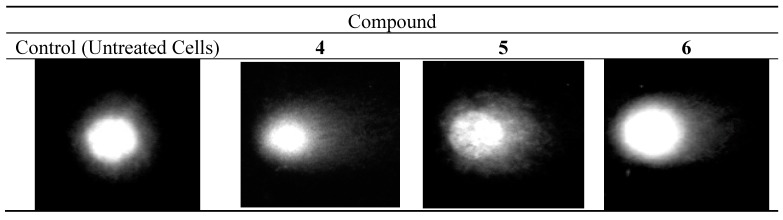
DNA comets obtained from MCF-7 cells treated for 24 h with **4**–**6** (single-cell electrophoresis).

The alkaline version of the DNA comet assay (pH > 13) was used to assess damage to DNA caused by compounds **4**–**6**. The method allows to specify both single- and double-strand DNA breaks and also damage to DNA nucleotides caused by the negative effects of oxidative stress. The technical details of the comet assay are described in the SI and the literature [56]. The results obtained from single-cell electrophoresis showed that compounds **4**, **5** and **6** damaged the DNA of both solid and leukemic cancer cells. This DNA-damaging activity contributed to the cytotoxic effect of the investigated compounds. Figure 6 shows images taken under a fluorescence microscope for the control and for MCF-7 cells treated with **4**, **5** and **6**, respectively. Analogous images for CCRF-CEM, HEPG2, and MDA-MB-231 cells treated with the tested complexes are shown in Figure S1 of the SI.

**Figure 7:**
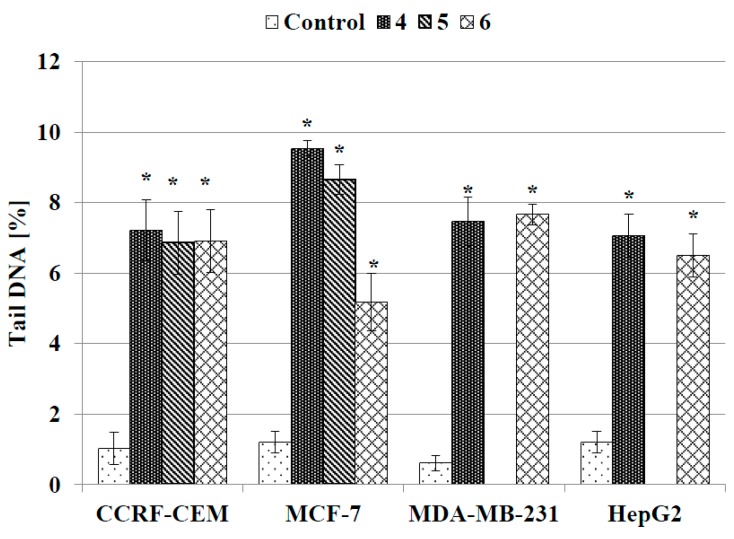
DNA content in the tail of the comet obtained from human cancer cells treated with compounds **4**, **5** and **6** for 24 h. The DNA tail fraction was estimated at the end of the incubation period by using an alkaline version of comet assay. Each experiment was done at least in triplicate. The results represent mean ± SEM of the data from three to four individual experiments, and for each analysis 100 randomly chosen comets were counted, * *p* < 0.05 *vs.* control.

As can be seen in Figure 6, supercoiled loops of DNA linked to the nuclear matrix of untreated control cells are seen as retaining a compact structure resembling a comet head, which after staining with fluorescence dye DAPI (4′,6-diamidino-2-phenylindole) emits very bright and intense fluorescence. On the contrary, DNA supercoils released from cells treated with the tested compounds are more relaxed and thus migrate behind the head, forming a comet tail with weaker fluorescence. Figure 7 shows quantified data on the DNA content in the tail of the comet obtained from human cancer cells treated with compounds **4**–**6**. Although the treated cells contained more tail DNA than the control cells, its amount did not exceed 10% and reached the highest value in MCF-7 cells incubated with compounds **4** and **5** (Figure 7). In the remaining treated cell lines the level of tail DNA was about 6%–8%, on average.

#### 2.3.5. Cell Cycle Analysis

The results of the comet assay were confirmed by flow cytometry analysis of the cell cycle of cancer cells incubated with complexes **4**–**6** for 24 h. The flow cytometry analysis revealed that the DNA damage observed in the comet assay is also reflected in the cancer cell’s mitotic cell cycle disturbances. An example of the analyzed DNA histograms is shown in Figure S2.

**Figure 8:**
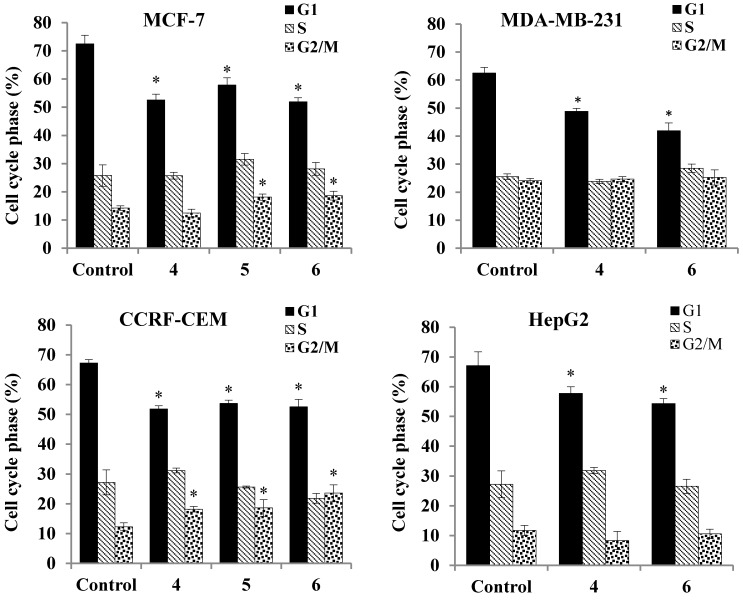
Cell cycle distribution of human cancer cells treated with compounds **4**, **5** and **6**. The cells were incubated with a IC_50_ concentration of the compounds for 24 h and then analyzed cytometrically. The results represent mean ± SEM of the data from three to four individual experiments, each done at least in sextuplicate. * *p* < 0.05 *vs.* control.

As shown in Figure 8, the inhibitory effect of the assayed compounds on the cell cycle results mostly in a moderate decrease in the percentage of cells in the G1 phase (15%–20%, on average). In the CCRF-CEM and MCF-7 cell lines, simultaneous accumulation of cells in the G2/M checkpoint is observed. On the contrary, no substantial differences were found in inhibitory effects on the cell cycle of MDA-MB-231 and HepG2 cells between the investigated compounds. Importantly, flow cytometry analysis also revealed an increase in the sub-G1 peak, including hypodiploid apoptotic cells in all of the treated cell lines (Figure S2). The most profound changes (*ca.* 20% increase) were found in MCF-7 cells treated with the most active compound **4** (Table 3). The presence of sub-G1 peaks on the histograms is an established indicator of the onset of apoptosis [57,58,59,60].

**Table 3:** Percentage of hypodiploid cancer cells calculated from the sub-G1 peak from DNA histograms estimated by cytometric analysis of the cell cycle. The values are the mean ± SEM of three independent experiments in 3–5 repeats each (* *p* < 0.05).

Cell Line	Cell Cycle Analysis Of DNA Histograms (Sub-G1 Fraction)			
	Control (Untreated Cell)	Compound		
		4	5	6
HepG2	1.95 ± 0.23	15.31 ± 1.64 *	-	12.46 ± 1 *
MDA-MB-231	2.39 ± 0.64	13.32 ± 1.39 *	-	5.0 ± 2.51
CCRF-CEM	2.81 ± 0.51	13.33 ± 1.37 *	6.18 ± 1.1	9.64 * ± 1.12
MCF-7	2.87 ± 0.9	18.58 ± 2.27 *	9.42 ± 1 *	10.48 ± 0.6 *

#### 2.3.6. Caspase Activation Assay

**Figure 9:**
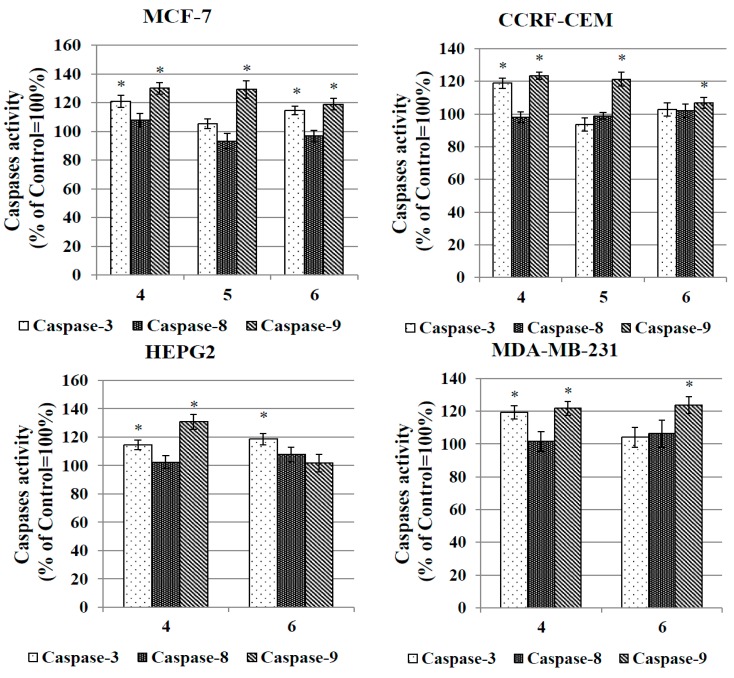
Relative activity of caspase-3, caspase-8 and capase-9 in human cancer cells treated with the IC_50_ concentrations of complexes **4**, **5** and **6** for 24 h. The results represent the mean ± SEM of the data from 3 to 4 individual experiments, each done at least in triplicate. * *p* < 0.05 *vs.* control.

In order to evaluate the pathway of apoptosis induced by the complexes, their ability to activate initiator caspases-8 and -9 and executioner caspase-3 was assessed. Activation of caspase-8 [61,62,63] is typical for the external (receptor) pathway of apoptosis, while activation of caspase-9 [64,65] is rather connected with the internal (mitochondrial) pathway. Both caspase-8 and caspase-9 can activate downstream caspase-3. Caspase-3 initiates apoptotic DNA fragmentation by proteolytically inactivating the DFF45/ICAD protein complex [65]. Figure 9 presents the data on the relative activity of caspases-8, -9 and -3 in cancer cell lines treated with complexes **4**–**6**.

Compounds **4**, **5** and **6** induced activation of caspases-9 and -3 but not of caspase-8. A *ca.* 25% increase in the activities of caspases-9 and -3 were observed in all cancer cell lines treated with compound **4** for 24 h. In the MCF-7 cells, activation of caspases-9 and -3 was also triggered by compounds **5** and **6**. The same two compounds were more selective and activated solely caspase-9 in CCRF-CEM (compound **5**) and MDA-MB-231 (compound **6**) cells. On the basis of the above results, it can be speculated that the gold complexes investigated here induce apoptosis of CCRF-CEM, HepG2, MCF-7 and MDA-MB-237 cells via the internal (mitochondrial) pathway.

#### 2.3.7. Evaluation of Antibacterial and Hemolytic Activity

Antibacterial activity of complexes **4**, **5**, **6** and reference compound Au(PPh_3_)Cl was expressed by minimal inhibitory concentration (MIC) values and tested against Gram-positive methicillin-sensitive *Staphylococcus aureus* (MSSA), methicillin-resistant *S. aureus* (MRSA) and Gram-negative *Escherichia coli* bacterial strains. The pertinent MIC data are presented in Table 4. In addition, compounds **4**–**6** were tested against four clinical isolates of the sensitive and resistant *S. aureus* strain (Table 4). The reference Au(PPh_3_)Cl complex revealed remarkable antibacterial (bactericidal) activity against whole tested Gram-positive *S. aureus* strains, including antibiotic-resistant MRSA pathogens. Its antibacterial potency was, however, lower against Gram-negative *E. coli* strains. Substitution of the Cl ligand in the reference Au(PPh_3_)Cl compound by either chromone (complexes **4** and **5**) or flavone (complex **6**) groups had a visible influence on the antibacterial activity profiles. Although high activity against Gram-positive *S. aureus* strains was retained for complexes **4** and **5**, the bactericidal effect of flavone derivative **6** against these strains was diminished. Moreover, compounds **4**–**6** lacked any antibacterial activity against Gram-negative *E. coli* strains.

In view of these data it can be speculated that in comparison to the Cl anion, chromone and flavone ligands significantly alter the uptake of compounds **4**–**6** into bacterial cells. As for the structural differences between the walls of Gram-positive *S. aureus* and Gram-negative *E. coli* bacterial strains, it can be hypothesized that the permeability of the latter with respect to complexes **4**–**6** is halted.

**Table 4:** *In vitro* antibacterial activity of complexes **4**–**6** and the Au(PPh_3_)Cl reference compound (MIC μg/mL).

Microorganism	MIC (μg/mL)			
	4	5	6	Au(PPh_3_)Cl
*S. aureus* subsp. *aureus* ATCC^®^ 29213™ (MSSA)	4	4	32	2
*S. aureus* subsp. *aureus* ATCC^®^ 43300 (MRSA)	2	2	2	1
*E. coli* ATCC^®^ 25922	>256	>256	>256	16
*E. coli* ATCC^®^*BAA-198*	>256	>256	>256	32
Clinical isolates: *S. aureus*				
(MSSA) 26/11	4	4	32	2
(MSSA) 30/11	4	4	32	2
(MRSA) 7/10	4	4	16	2
(MRSA) 41/12	4	4	32	2

MIC value of the reference compound, penicillin G against *S. aureus* ATCC 29213–2 µg/mL.

Complexes **4**–**6** were examined *in vitro* for hemolytic activity. No hemolytic activity of **4**–**6** was observed on human erythrocytes.

## 3. Experimental Section

### 3.1. General Information

All preparations were carried out using standard Schlenk techniques. Chromatographic separations were carried out using silica gel 60 (Sigma-Aldrich, 230–400 mesh ASTM, St. Louis, MO, USA). Dichloromethane was freshly distilled from CaH_2_ handled under nitrogen. Other solvents were of reagent grade and were used without prior purification. Alkyne substrates were obtained according to the literature method [50,53]. All other chemicals were purchased from the Sigma-Aldrich Chemical Co. The NMR spectra were recorded on a AV600 Kryo (600 MHz) spectrometer (Bruker, Bremen, Germany). Chemical shifts are reported in δ (ppm) using residual CHCl_3_ (^1^H δ 7.26 ppm, ^13^C δ 77.00 ppm) and DMSO (^1^H δ 2.50 ppm) as the references. Mass spectra were recorded using ES methods on a Varian 500-MS iT mass spectrometer (Agilent, Santa Clara, CA, USA). IR spectra were recorded on a FTIR Nexus Nicolet apparatus (Nicolet, Madison, WI, USA). Microanalyses were determined by the Analytical Services of the Polish Academy of the Sciences (Łódź, Poland).

### 3.2. Chemistry

#### 3.2.1. Synthesis of **4**

In a 40 mL Schlenk tube, alkynyl chromone **1** (100 mg, 0.3 mmol) was dissolved in dry and degassed dichloromethane (35 mL). The resultant solution was purged with argon for 15 min at room temperature. To this solution were added Au(PPh_3_)Cl (149 mg, 0.3 mmol) and diisopropylamine (1.5 mL, 11 mmol). The reaction mixture was protected against light and stirred at 40 °C for 24 h. After cooling to r.t. the reaction mixture was evaporated to dryness. The residue was subjected to flash chromatography on SiO_2_ (eluent: CHCl_3_/MeOH/Et_3_N, 50:2:0.5). The analytically pure product **4** was obtained after recrystallization from dichloromethane/*n*-hexane mixture. Colorless solid. Yield: (187 mg, 79%); ^1^H-NMR (600 MHz, CDCl_3_): δ = 8.324 (d, *J*_H,H_ = 2.4 Hz, 1H, H5), 7.91 (s, 1H, H2), 7.65 (dd, *J*_H,H_ = 9.0 Hz, 2.4 Hz, 1H, H7), 7.52–7.41 (m, 15H, Ph), 7.28 (d, *J*_H,H_ = 9.0 Hz, 1H, H8), 5.64 (bs, 1H, NH), 4.15 (d, *J*_H,H_ = 4.8 Hz, 2H, NHCH_2_), 2.78 (t, *J*_H,H_ = 6.6 Hz, 2H, CH_2_), 2.49 (t, *J*_H,H_ = 6.6 Hz, 2H, CH_2_) ppm. ^31^P{^1^H}-NMR (243 MHz, CDCl_3_): δ = 42.10 (s) ppm. ^13^C{^1^H}-NMR (150 MHz, CDCl_3_): δ = 176.6, 176.5, 171.5, 171.0, 155.2, 153.7, 136.4, 136.2, 134.2 (d, *J*_P,C_ = 54 Hz), 131.6 (bs), 129.14(d, *J*_P,C_ = 42 Hz), 128.4, 128.3, 125.2, 125.1, 123.1, 122.9, 120.14, 120.12, 118.3, 118.2, 71.3, 34.3, 30.6, 29.6, 29.0, 26.9, 22.1, 21.9. MS (+ESI, 70eV): *m*/*z* = 792 (MH^+^), 721 (Au(PPh_3_)_2_)^+^). FTIR (KBr, cm^−1^): 3285, 3054, 2925, 2853, 2119 (C≡C very weak), 1637 (CO), 1605 (CO), 1437. Anal. Calcd for C_33_H_26_NO_3_PBrAu: C, 50.02; H, 3.31. Found: C, 50.01; H, 3.14%.

#### 3.2.2. Synthesis of **5**

In a 40 mL Schlenk tube, alkynyl chromone **2** (98 mg, 0.21 mmol) was dissolved in dry and degassed dichloromethane (35 mL). The resultant solution was purged with argon for 15 min at room temperature. To this solution were added Au(PPh_3_)Cl (104 mg, 0.21 mmol) and diisopropylamine (1 mL, 7 mmol). The reaction mixture was protected against light and stirred at 40 °C for 24 h. After cooling to r.t. the reaction mixture was evaporated to dryness. The residue was subjected to flash chromatography on SiO_2_(eluent: CHCl_3_/MeOH/Et_3_N, 50:2:0.5). The analytically pure product **5** were obtained after recrystallization from dichloromethane/*n*-hexane mixture. Orange solid. Yield: (130 mg, 67%); ^1^H-NMR (600 MHz, DMSO-*d*_6_): δ = 8.18 (s, 1H, H2), 8.10 (t, *J*_H,H_ = 5.4 Hz, 1H, NH), 8.05 (d, *J*_H,H_ = 1.8 Hz, 1H, H5), 7.90 (dd, *J*_H,H_ = 8.7 Hz, 1.8 Hz, 1H, H7), 7.63–7.49 (m, 16H, Ph, H8), 7.07 (d, *J*_H,H_ = 16.2 Hz, 1H, FcHC=CH-), 6.91 (d, *J*_H,H_ = 16.2 Hz, 1H, FcHC=CH-), 4.60 (pseudo-t, *J*_H,H_ = 1.8 Hz, 2H, α-C_5_H_4_), 4.33 (pseudo-t, *J*_H,H_ = 1.8 Hz, 2H, β-C_5_H_4_), 4.14 (s, 5H, C_5_H_5_), 3.85 (d, *J*_H,H_ = 5.4 Hz, 2H, NHCH_2_), 2.61 (t, *J*_H,H_ = 7.2 Hz, 2H, CH_2_), 2.36 (t, *J*_H,H_ = 7.2 Hz, 2H, CH_2_) ppm. ^31^P{^1^H}-NMR (243 MHz, DMSO-*d*_6_): δ = 41.71 (s) ppm. ^13^C{^1^H}-NMR (150 MHz, CDCl_3_): δ = 176.5, 170.7, 154.9, 153.5, 134.9, 134.0 (d, *J*_P,C_ = 54 Hz), 132.0 (d, *J*_P,C_ = 12 Hz), 130.9, 129.7(d, *J*_P,C_ = 48 Hz), 129.2, 128.7, 124.1, 123.4, 122.8, 121.3, 118.8, 82.8, 69.6, 69.17, 69.14, 67.1, 34.3, 33.7, 29.2, 26.4, 24.8, 21.5. MS (+ESI, 70eV): *m*/*z* = 923 (M^+^), 721 (Au(PPh_3_)_2_)^+^). FTIR (KBr, cm^−1^ ): 3075, 3054, 2924, 2852, 2120 (C≡C very weak), 1637 (CO), 1605 (CO), 1487, 1437. Anal. Calcd for C_45_H_37_NO_3_PFeAu: C, 58.52; H, 4.04. Found: C, 58.62; H, 4.44%.

#### 3.2.3. Synthesis of **6**

In a 40 mL Schlenk tube, alkynyl chromone **3** (138 mg, 0.56 mmol) was dissolved in dry and degassed dichloromethane (35 mL). The resultant solution was purged with argon for 15 min at room temperature. To this solution were added Au(PPh_3_)Cl (278 mg, 0.56 mmol) and diisopropylamine (2.8 mL, 20 mmol). The reaction mixture was protected against light and stirred at 40 °C for 24 h. After cooling to r.t. the reaction mixture was evaporated to dryness. To purify product **6** two subsequent chromatographic purifications on SiO_2_ were necessary. The first with *n*-hexane/ethyl acetate, (3:2) as eluting mixture and the second with toluene/ethyl acetate (4:1) as eluting mixture respectively. The analytically pure product **6** were obtained after recrystallization from dichloromethane/*n*-hexane mixture. Colorless solid. Yield: (193 mg, 49%); ^1^H-NMR (600 MHz, CDCl_3_): δ = 8.358 (d, *J*_H,H_ = 1.8 Hz, 1H, H5), 7.90 (dd, *J*_H,H_ = 7.2 Hz, 1.2 Hz, 2H, Ph), 7.79 (dd, *J*_H,H_ = 8.4 Hz, 1.8 Hz, 1H, H7), 7.58–7.43 (m, 19H, Ph, H8), 6.77 (s, 1H, H3) ppm. ^31^P{^1^H}-NMR (243 MHz, CDCl_3_): δ = 42.23 (s) ppm. ^13^C{^1^H}-NMR (150 MHz, CDCl_3_): δ = 177.7, 163.0, 154.9, 137.6, 134.2 (d, *J*_P,C_ = 54 Hz), 131.9, 131.5 (d, *J*_P,C_ = 12 Hz), 131.4, 129.9, 129.5, 129.19 129.17 (d, *J*_P,C_ = 42 Hz), 129.0, 126.2, 123.8, 122.5, 117.8, 107.6. MS (+EI, 70eV): 721 (Au(PPh_3_)_2_)^+^), 705 (M^+^). FTIR (KBr): 3067, 2923, 2854, 2117(C≡C), 1644 (CO), 1610 (CO), 1563, 1478, 1434, 1355. Anal. Calcd for C_35_H_24_O_2_PAu: C, 59.67; H, 3.43. Found: C, 59.39; H, 3.52%.

### 3.3. X-ray Crystallography

Orange crystals of **5**·CH_2_Cl_2_·0.5 C_4_H_10_ were obtained by the slow evaporation of a CH_2_Cl_2_/*n*-butane solution, while colorless crystals of **6** were grown by the slow diffusion of *n-*hexane into a CH_2_Cl_2_ solution of the complex. Data were collected on a Stoe Image Plate Diffraction system equipped with a Φ circle goniometer, using Mo-Kα graphite monochromatic radiation (λ = 0.71073 Å) with Φ range 0–200°. The structures were solved by direct methods using the program SHELXS-97, while the refinement and all further calculations were carried out using SHELXL-97 [66]. The H-atoms were included in calculated positions and treated as riding atoms using the SHELXL default parameters. The non-H atoms were refined anisotropically, using weighted full-matrix least-square on *F*^2^. Crystallographic details for **5**·CH_2_Cl_2_·0.5 C_4_H_10_ and **6** are summarized in Table S1. Figure 1 and Figure 2 were drawn with ORTEP [67] and Figure 3 with Mercury [68]. CCDC 1061082 **5**·CH_2_Cl_2_·0.5 C_4_H_10_ and 1061083 **6** contain the supplementary crystallographic data for this paper. These data can be obtained free of charge from The Cambridge Crystallographic Data Centre via www.ccdc.cam.ac.uk/data_request/cif.

### 3.4. Biology

#### 3.4.1. Cell Lines

Three adherent tumor cell lines: human hepatocellular carcinoma (HepG2; ATCC HB-8065™), estrogen-responsive adenocarcinoma (MCF-7; ATCC HTB-22™) and estrogen-unresponsive adenocarcinoma (MDA-MB-231; ATCC HTB-26™) supplied by ATCC (Rockville, Manassas, VA, USA) were used in all experiments. Suspension T lymphoblast like polymorph cell line (CCRF-CEM) was kindly donated by Prof. G. Bartosz from Department of Molecular Biophysics, University of Lodz (Poland). Additionally adherent human endothelial cell line HUVEC-ST supplied by ATCC (Rockville, Manassas, VA, USA) were used as a model for normal cells in the MTT assay.

#### 3.4.2. Cell Culture

Adherent cell lines (HepG2, MCF-7 and MDA-MB-231) were cultured as a monolayer in Dulbecco’s modified Eagle’s medium (DMEM) (Sigma, Lonza, Verviers, Belgium), supplemented with 10% (*v*/*v*) fetal bovine serum (Gibco, Grand Island, New York, NY, USA), penicillin-streptomycin (10 mg/mL) 1% (*v*/*v*) (Gibco) under a 100% humidified atmosphere of 5% CO_2_ and 95% air, at 37 °C. Exponential growth of cells was maintained by their regular passaging at 90% confluence three times a week using 0.025% trypsin/EDTA. CCRF-CEM cells were grown in suspension in RPMI 1640 medium enriched with 10% (*v*/*v*) fetal bovine serum (Gibco), penicillin-streptomycin (10 mg/mL) 1% (*v*/*v*) (Gibco) under the culture conditions described above. Cell cultures, after centrifugation at 400 rpm for 5 min, were resuspended at a density of 2 × 10^5^ viable cells/mL. Periodically, all cell lines were routinely screened for mycoplasma contamination.

#### 3.4.3. Evaluation of Cytotoxic Activity (MTT Assay)

Anticancer activity of complexes **4**, **5** and **6** was evaluated *in vitro* on the basis of their ability to inhibit the proliferation of HepG2, MCF-7, MDA-MB-231 and CCRF-CEM cancer cells. For this purpose a standard MTT microplate assay with 3-[4,5-dimethylthiazol-2-yl)-2,3-diphenyltetrazolium bromide (Sigma Aldrich) was used. Cells were seeded a day before the experiment onto a 96-well microplate at the density up to 12 × 10^3^ cells/well (depending on the cell line) and exposed to investigated compounds, resolved in an appropriate culture medium in a concentration range of 0.5–120 μM, for 24 h. At the end of incubation the medium was replaced with a new one devoid of organometallic derivatives. The cells were grown for additional 48 h (post-incubation culture in a drug-free medium). At this time point the number of viable cells in each well was estimated by the MTT test. 50 µL of MTT (5 mg/mL final concentration) was added to each microplate well and the cells were incubated for 4 h in a CO_2_ incubator. Then MTT solution was discarded and a 100 µL DMSO/well was added in order to dissolved formazan crystals formed during the reduction of MTT by mitochondrial dehydrogenases of metabolically active cells. Absorbance of purple formazan solution was measured spectrophotometrically at 570 nm. Nonadherent CCRF-CEM were centrifuged for 5 min at 800 rpm each time before replacing an old solution with new one. Cytotoxicity of the assayed complexes was evaluated on the basis of their IC_50_ concentrations that reduced cell viability by 50% compared with that of untreated control cells, arbitrary taken as 100%. Moreover, similar evaluation was conducted for human endothelial cell line (HUVEC-ST) used as a model for normal cells.

#### 3.4.4. Thioredoxin Reductase Inhibition Assay

To assess the enzymatic activities of the thioredoxin reductase (TrxR) activity colorimetric assay kit (Bio Vision, Milpitas, CA, USA) was used according to the manufacturer’s protocol. The method is based on the ability of TrxR to catalyze the reduction of 5,5′-dithiobis-2-nitrobenzoic acid (DTNB) with NADPH to 5-thio-2-nitrobenzoic acid (TNB2), which generate a strong yellow color with maximum at 412 nm). Since in crude biological samples other enzymes, such as glutathione reductase and glutathione peroxidase, can also reduce DTNB, therefore, TrxR specific inhibitor is utilized to determine TrxR specific activity. Two assays are performed: the first measurement is of the total DTNB reduction by the sample, and the second one is the DTNB reduction by the sample in the presence of the TrxR specific inhibitor. The difference between the two results is the DTNB reduction by TrxR. Cells were seeded onto 35 mm dishes at the density of 50 × 10^4^ and cultured for 24 h in drug-free medium. Then tested complexes were added at IC_50_ concentration and the cells were incubated with the compounds for another 24 h. At the end of incubation the cells were homogenized in a cold Assay Buffer and centrifuged at 10,000 rpm for 15 min at 4 °C. Supernatant was collected. Te each well was added 50 µL of lysed cell sample and 40 µL of reaction mix containing assay buffer, DTNB and NADPH and mixed well. For testing background enzyme activity, to the appropriate well was added 10 µL of TrxR inhibitor. The reaction was measured by determining the change in absorbance at 412 nm using the microplate reader in 0–20 min time of incubation.

#### 3.4.5. DNA Comet Assay

Alkaline version of DNA comet assay (pH > 13) was used to assess damage to DNA caused by Au-compound treatment. The method allows to specify both single- and double-strand DNA breaks and also damage to the DNA nucleotides caused by the negative effects of oxidative stress. Single cells were embedded in an agarose gel, then treated with lysis buffer, subjected to electrophoresis, stained with a 4′,6-diamidino-2-phenylindole (DAPI) solution and analyzed under a fluorescent microscope. Image of a single cell resembles the shape of a comet in which the circular head and tail can be distinguished. The head of the comet consists of a undamaged high molecular weight DNA which in the conditions used does not migrate during the electrophoresis into agarose gel. Damaged DNA of low molecular weight migrates in the tail of the comet in the direction of the anode. In the experiments the cells were seeded on 35 mm dishes and cultured for 24 h in drug-free medium. Then Au-compounds were added at IC_50_ concentration and the cells were incubated with them for another 24 h. At the end of incubation, cells were released from the monolayer by trypsinization, resuspended in 0.5 mL PBS and centrifuged for 5 min at 4 °C at 2500 rpm. Then the cell suspension was added to the solution of low melting point agarose. A small amount of this suspension was applied to a glass slide pre-coated with a standard agarose solution and covered with a coverslip. After the agarose solidified, the cover slip was removed and the preparations were incubated for a minimum of 1 h with a lysis buffer (2.5 mol/L NaCl, 100 mmol/L Na_2_-EDTA, 10 mmol/L Tris, 1% Triton X-100) to release the DNA from the cells. Next the slides with cells were washed three times with developing buffer (1 mmol/L Na_2_-EDTA, 300 mmol/L NaOH), incubated therein for 20 min and subjected to electrophoresis in an electrophoretic buffer for 20 min (electric field 29 V and 30 MA). After electrophoresis, the slides were allowed to dry and then stained with DAPI solution (2 μg/mL). DNA comet analysis was performed ubder a Nikon Eclipse fluorescence microscope (Nikon, Tokyo, Japan), equipped with a 4910COHU video camera (Cohu, Inc., San Diego, CA, USA) and connected to a computer with installed software Lucia-Comet v.4.51 (Laboratory Imaging, Praha, Czech Republic). For each analysis 100 comets from each formulation were counted.

#### 3.4.6. Cell Cycle Analysis

To assess the influence of investigated compounds on proliferation of treated cells the cell cycle distribution was analysed on Becton Dickinson LSR II cytometer. Cells were plated on a 35 mm dish at a density of 1 × 10^6^/mL and cultured for 24 h in drug-free medium. After then, gold organometallics compounds was added (IC_50_ concentration) and the incubation was continued for additional 24 h. At the end of the treatment, the cell monolayer was washed with PBS and then trypsinized. The harvested cell suspension was centrifuged at 4 °C for 5 min and 1000 rpm. The supernatant was discarded, the cell pellet was washed with a PBS (Ca^2+^, Mg^2+^-free) and the cells were resuspended in 200 μL of PBS. After then, the cells were fixed in 70% cold ethanol (−20 °C) and stored at this temperature until analysis. Before cytometric measurements, cells were centrifuged at 4 °C for 5 min and 2000 rpm. The supernatant was poured off and the cell pellet was washed with cold PBS solution. After next centrifugation cells were resuspended in 500 μL HBSS buffer containing 1 M Tris HCl, pH 7,5 (final concentration 10 mM), 1 M MgCl_2_ (final concentration 5 mM), RNAse A 10 mg/mL (final concentration 10 μg/mL) and propidium iodide (4 mg/1 mL DMSO, final concentration 5 μM). The cells were incubated for 30–60 min at 37 °C and proceeded for a cytometric measurement. Analysis of DNA histogram was performed by FLOWJO, LLC data analysis software (Ashland, OR, USA).

#### 3.4.7. Caspases Activation Assay

##### Caspase-8 and Caspase-9 Activities

Colorimetric assay kits according to the manufacturer’s protocol (R & D Systems, Minneapolis, MN, USA) were used to assess the enzymatic activities of caspases-8 and -9. Cells that have been exposed to the appropriate IC_50_ parameter of investigated organometallic compounds were seeded on 35 mm dishes at the density of 50 × 10^4^ and cultured for 24 h in drug-free medium. Then complexes **4**–**6** were added at IC_50_ concentration and the cells were incubated with the compounds for another 24 h. At the end of incubation, cells were released from the monolayer by trypsinization and lysed in the supplied lysis buffer. The cell lysate was centrifuged at 10,000 rpm for 1 min. The supernatant was collected and incubated at 37 °C with the supplied reaction buffer containing dithiothreitol and substrates caspase-specific peptide that is conjugated to the color reporter molecule p-nitroaniline (pNA). The cleavage of the peptide by the caspase releases the chromophore pNA, which absorbance (405 nm) is directly proportional to the level of caspase enzymatic activity in the cell lysate. The reaction was measured by determining the change in absorbance at 405 nm using the microplate reader.

##### Caspase-3 Activity

To assess the enzymatic activities of the caspase-3 fluorometric assays were used according to the manufacturer’s protocol (Molecular Probes, Minneapolis, MN, USA) in a kit for caspase activity. Caspase activity assay kits were purchased from Molecular Probes. Cells that have been exposed to the appropriate IC_50_ parameter of investigated organometallic compounds were seeded dishes of 35 mm at the density of 50 × 10^4^ and cultured for 24 h in drug-free medium. Then assayed compounds were added in concentrations of examined IC_50_ and incubated for another 24 h. After incubation, cells were released from monolayer by trypsinization and lysed in the supplied lysis buffer. The cell lysate was centrifuged at 5000 rpm for 5 min. The supernatants were collected and incubated with the supplied reaction buffer containing dithiothreitol and substrates at 37 °C. The reaction was measured by determining the change in fluorescence (excitation/emission ~496/520 nm) using excitation and emission filters or settings appropriate for fluorescein.

#### 3.4.8. Statistical Analysis

All data are expressed as a mean ± SEM. An ANOVA analysis of variance with a Tuckey post hoc test were used for multiple comparisons. All statistics were calculated using a statistical program STATISTICA (StatSoft, Tulsa, OK, USA). A *p* value of <0.05 was considered significant.

#### 3.4.9. Cellular Uptake and AAS Measurements

For cellular uptake studies MCF-7 cells were grown until at least 70% confluency in 75 cm^2^ cell culture flasks. Stock solutions of complexes **4**–**6** in DMF were freshly prepared and diluted with cell culture medium to the desired concentrations (final DMF concentration: 0.1% *v*/*v*, final complex concentration: 3.0 μM). The cell culture medium of the cell culture flasks was replaced with 10 mL of the cell culture medium solutions containing the complexes and the flasks were incubated for 1, 4 and 8 h at 37 °C/5% CO_2_. Afterwards the medium was removed and the cells were washed with phosphate buffered saline pH 7.4. After trypsinization, cell pellets were isolated by centrifugation, resuspended in 300 µL twice distilled water, lysed by ultrasonication and appropriately diluted using twice distilled water. The gold content of the samples was determined by atomic absorption spectroscopy (AAS, see below) and the protein content of separate aliquots by the Bradford method. AAS measurements were performed using a contrAA 700 high resolution continuum source atomic absorption spectrometer (HRCS-AAS) for the quantification of gold. Gold was detected at a wavelength of 242.7950 nm. Standards for calibration purposes were prepared in a matrix matched manner (cellular matrix) using a standard stock solution of **4**. Triton X-100 (20 µL, 1%) and ascorbic acid (20 µL, 1%) were added to each 200 µL sample or standard solution. A volume of 25 µL thereof was injected into the graphite tubes. Drying, pyrolysis, and atomization in the graphite furnace were performed according to the conditions previously with minor modifications [69]. The mean absorbances of triplicate injections were used throughout the study.

##### Antibacterial Activity

Antibacterial activity of compounds **4**–**6** and reference ClAuPPh_3_ was tested by liquid microdilution method. The antimicrobial spectrum of **4**–**6** compounds and ClAuPBn_3_ was evaluated by the minimal inhibitory concentrations (MIC) method using the serial two-fold dilution method under standard conditions as described in the Clinical and Laboratory Standards Institute (CLSI) reference method M07-A8 [70]. A Gram-positive bacterial strains, *Staphylococcus aureus* ATCC^®^ 29213 (sensitive to methicillin, (MSSA)), *Staphylococcus aureus* ATCC^®^ 43300 (resistant to methicillin, (MRSA)) and Gram-negative, *Escherichia coli* ATCC^®^ 25922™, *Escherichia coli* ATCC^®^ BAA-198 were used. Four clinical isolates of *Staphylococcus aureus* (MSSA and MRSA) obtained from Department of Pharmaceutical Microbiology of Medical University of Warsaw, Poland were also used. The clinical isolates were collected from various patients hospitalized in several clinics. Bacterial strains were cultivated on tryptic soy agar (TSA) according to ATCC recommendation. All strains were incubated for 24 h at 37 °C. Reference method (broth microdilution susceptibility test) was as follows: assayed compounds (**4**–**6** and Au(PPh_3_)Cl) were dissolved in DMSO. A series of the twofold **4**–**6** and Au(PPh_3_)Cl compounds dilutions were diluted with cation–adjusted Mueller-Hinton broth (CAMHB). 95 µL aliquots were dispensed into microdilution sterile plates (Mar-Four). Then, 5 µL of bacteria inoculum, containing 5 × 10^4^ CFU·mL^−1^, was added. The final concentration of **4**–**6** and Au(PPh_3_)Cl compounds ranged from 512 to 0.5 µg·mL^−1^ all in two-fold dilution steps. The experiments for each sample were conducted in triplicate. Penicillin G were used as controls (from 8–0.15 µg·mL^−1^). The plates were incubated at 37 °C for 18 to 24 h depending on bacterial strain. Results were obtained with the use of Spectrostar Omega (BMG Labtech, Offenburg, Germany), absorbance was measured at λ = 540 nm and λ = 595 nm. MIC was defined as the lowest drug concentration that reduced growth by 100%. MIC value of the reference compound, penicillin G against *S. aureus* ATCC^®^ 29213–2.0 µg·mL^−1^.

##### Hemolysis

Hemolysis test was evaluated following the literature method [71]. Red blood cells were obtained from healthy donor. The erythrocytes prepared in the PBS were suspended to corresponding to a hematocrit of 1% in solutions containing complexes **4**–**6** in final concentrations from 0.001 to 0.2 mM and incubated for 30 min at 23 °C. After centrifugation (1000 rpm, 5 min) the absorbance of supernatant was measured at 540 nm (Jasco V-630, Jasco, Tokyo, Japan). A value of 100% hemolysis was determined by incubation of erythrocytes with double-distilled water (30 min at 23 °C).

## 4. Conclusions

In summary, three new triphenylophosphine gold(I)-alkynyl chromones were obtained and characterized. In the solid state, chromone moieties in complex **6** alligned to form layers which were only separated by 3.34Å, as determined by single-crystal X-ray structure analyses. Anticancer and antibacterial activity of the complexes was evaluated *in vitro* against HepG2, MCF-7, MDA-MB-231 and CCRF-CEM human cancer cells and *S. aureus* (MSSA), *S. aureus* (MRSA) and *E. coli* bacterial strains, respectively. The compounds were taken up by cancer cells, as shown by the AAS experiments, and showed cytotoxic activity at low micromolar concentrations. Molecular mechanisms accounted for the observed anticancer activity involved: thioredoxin reductase (TrxR) inhibition, initiator caspase-9 and executioner caspase-3 activation and genotoxic effect. In general, the obtained results showed pro-apoptic activity of the investigated gold complexes and suggested that the mitochondria were their major cellular target. Gold(I) complexes showed strong antibacterial activity against Gram-positive *S. aureus* strains, including methicillin-resistant pathogens. On the contrary, they were devoid of activity against Gram-negative *E. coli* bacterial strains. This can be explained by hindered uptake of complexes through the cell wall of Gram-negative bacteria. The case of Au-Fe bimetallic complex **5** also demonstrates that the presence of two different metals in one molecular scaffold does not immediately benefit in enhanced biological activity.

## Supplementary Materials

Supplementary materials can be accessed at: http://www.mdpi.com/1420-3049/20/11/19647/s1.

## References

[1] Rosenberg B., Van C.L., Krigas T. (1965). Inhibition of Cell Division in *Escherichia coli* by Electrolysis Products from a Platinum Electrode. Nature.

[2] Lippert B. (1999). Cisplatin: Chemistry and Biochemistry of a Leading Anticancer Drug.

[3] Berners-Price S.J. (2011). Activating Platinum Anticancer Complexes with Visible Light. Angew. Chem. Int. Ed..

[4] Allardyce C.S., Dyson P.J., Ellis D.J., Heath S.I. (2001). [Ru(η^6^-p-cymene)Cl_2_(pta)] (pta = 1,3,5-triaza-7-phosphatricyclo-[3.3.1.1]decane): A water soluble compound that exhibits pH dependent DNA binding providing selectivity for diseased cells. Chem. Commun..

[5] Pacor S., Zorzet S., Cocchietto M., Bacac M., Vadori M., Turrin C., Gava B., Castellarin A., Sava G. (2004). Intratumoral NAMI-A Treatment Triggers Metastasis Reduction, Which Correlates to CD44 Regulation and Tumor Infiltrating Lymphocyte Recruitment. J. Pharmacol. Exp. Ther..

[6] Hartinger C.G., Jakupec M.A., Zorbas-Seifried S., Groessl M., Egger A., Berger W., Zorbas H., Dyson P.J., Keppler B.K. (2008). KP1019, A New Redox-Active Anticancer Agent—Preclinical Development and Results of a Clinical Phase I Study in Tumor Patients. Chem. Biodivers..

[7] Debreczeni J.E., Bullock A.N., Atilla G.E., Williams D.S., Bregman H., Knapp S., Meggers E. (2006). Ruthenium Half-Sandwich Complexes Bound to Protein Kinase Pim-1. Angew. Chem. Int. Ed..

[8] Vessières A., Top S., Beck W., Hillard E., Jaouen G. (2006). Metal complex SERMs (selective oestrogen receptor modulators). The influence of different metal units on breast cancer cell antiproliferative effects. Dalton Trans..

[9] Citta A., Folda A., Bindoli A., Pigeon P., Top S., Vessières A., Salmain M., Jaouen G., Rigobello M.P. (2014). Evidence for Targeting Thioredoxin Reductases with Ferrocenyl Quinone Methides. A Possible Molecular Basis for the Antiproliferative Effect of Hydroxyferrocifens on Cancer Cells. J. Med. Chem..

[10] Rubbiani R., Wahrig B., Ott I. (2014). Historical and biochemical aspects of a seventeenth century gold-based aurum vitae recipe. J. Biol. Inorg. Chem..

[11] Koch R. (1890). Ueber bacteriologische Forschung. Dtsch. Med. Wochenstr..

[12] Forestier J. (1935). Rheumatoid arthritis and its treatment by gold salts: The results of six years’ experience. J. Lab. Clin. Med..

[13] Simon T.M., Kunishima D.H., Vibert G.J., Lorber A. (1981). Screening Trial with the Coordinated Gold Compound Auranofin Using Mouse Lymphocytic Leukemia P388. Cancer Res..

[14] Mirabelli C.K., Johnson R.K., Sung C.M., Faucette L., Muirhead K., Crooke S.T. (1985). Evaluation of the *in Vivo* Antitumor Activity and *in Vitro* Cytotoxic Properties of Auranofin, a Coordinated Gold Compound, in Murine Tumor Models. Cancer Res..

[15] Ott I. (2009). On the medicinal chemistry of gold complexes as anticancer drugs. Coord. Chem. Rev..

[16] Liu W., Gust R. (2013). Metal *N*-heterocyclic carbene complexes as potential antitumor metallodrugs. Chem. Soc. Rev..

[17] Hackenberg F., Tacke M. (2014). Benzyl-substituted metallocarbene antibiotics and anticancer drugs. Dalton Trans..

[18] Cisnetti F., Gautier A. (2013). Metal/*N*-Heterocyclic Carbene Complexes: Opportunities for the Development of Anticancer Metallodrugs. Angew. Chem. Int. Ed..

[19] Glišić B.D., Djuran M.I. (2014). Gold complexes as antimicrobial agents: An overview of different biological activities in relation to the oxidation state of the gold ion and the ligand structure. Dalton Trans..

[20] Tacke M. (2015). Benzyl-substituted carbene–metal complexes: Potential for novel antibiotics and anticancer drugs?. J. Organomet. Chem..

[21] Bagowski C.P., You Y., Scheffler H., Vlecken D.H., Schmitz D.J., Ott I. (2009). Naphthalimide gold(I) phosphine complexes as anticancer metallodrugs. Dalton Trans..

[22] Ott I., Qian X., Xu Y., Vlecken D.H.W., Marques I.J., Kubutat D., Will J., Sheldrick W.S., Jesse P., Prokop A. (2009). A Gold(I) Phosphine Complex Containing a Naphthalimide Ligand Functions as a TrxR Inhibiting Antiproliferative Agent and Angiogenesis Inhibitor. J. Med. Chem..

[23] Wang V.-H., Shih W.-C., Chang H.C., Kuo Y.-Y., Hung W.-C., Ong T.-G., Li W.-S. (2011). Preparation and Characterization of Amino-Linked Heterocyclic Carbene Palladium, Gold, and Silver Complexes and Their Use as Anticancer Agents That Act by Triggering Apoptotic Cell Death. J. Med. Chem..

[24] Rubbiani R., Salassa L., de Almeida A., Casini A., Ott I. (2014). Cytotoxic Gold(I) *N*-heterocyclic Carbene Complexes with Phosphane Ligands as Potent Enzyme Inhibitors. ChemMedChem..

[25] Bertrand B., de Almeida A., van der Burgt E.P.M., Picquet M., Citta A., Folda A., Rigobello M.P., le Gendre P., Bodio E., Casini A. (2014). New Gold(I) Organometallic Compounds with Biological Activity in Cancer Cells. Eur. J. Inorg. Chem..

[26] Arcau J., Andermark V., Rodrigues M., Giannicchi I., Pérez-Garcia L., Ott I., Rodríguez L. (2014). Synthesis and Biological Activity of Gold(I) *N*-Heterocyclic Carbene Complexes with Long Aliphatic Side Chains. Eur. J. Inorg. Chem..

[27] Hickey J.L., Ruhayel R.A., Barnard P.J., Baker M.V., Berners-Price S.J., Filipovska A. (2008). Mitochondria-Targeted Chemotherapeutics: The Rational Design of Gold(I) *N*-Heterocyclic Carbene Complexes That Are Selectively Toxic to Cancer Cells and Target Protein Selenols in Preference to Thiols. J. Am. Chem. Soc..

[28] Wetzel C., Kunz P.C., Kassack M.U., Hamacher A., Böhler P., Watjen W., Ott I., Rubbiani R., Spingler B. (2011). Gold(I) complexes of water-soluble diphos-type ligands: Synthesis, anticancer activity, apoptosis and thioredoxin reductase inhibition. Dalton Trans..

[29] Balasingham R.G., Williams C.F., Mottram H.J., Coogan M.P., Pope S.J.A. (2012). Gold(I) Complexes Derived from Alkynyloxy-Substituted Anthraquinones: Syntheses, Luminescence, Preliminary Cytotoxicity, and Cell Imaging Studies. Organometallics.

[30] Meyer A., Gutiérrez A., Ott I., Rodríguez L. (2013). Phosphine-bridged dinuclear gold(I) alkynyl complexes: Thioredoxin reductase inhibition and cytotoxicity. Inorg. Chim. Acta..

[31] Chui C.-H., Wong R.S.-M., Gambari R., Cheng G.Y.-M., Yuen M.C.-W., Chan K.-W., Tong S.-W., Tong F.-Y., Lau F.-Y., Lai P.B.-S. (2009). Antitumor activity of diethynylfluorene derivatives of gold(I). Bioorg. Med. Chem..

[32] Schuh E., Valiahdi S.M., Jakupec M.A., Keppler B.K., Chiba P., Mohr F. (2009). Synthesis and biological studies of some gold(I) complexes containing functionalised alkynes. Dalton Trans..

[33] Vergara E., Cerrada E., Casini A., Zava O., Laguna M., Dyson P.J. (2010). Antiproliferative Activity of Gold(I) Alkyne Complexes Containing Water-Soluble Phosphane Ligands. Organometallics.

[34] Meyer A., Bagowski C.P., Kokoschka M., Stefanopoulou M., Alborzinia H., Can S., Vlecken D.H., Sheldrick W.S., Wölfl S., Ott I. (2012). On the Biological Properties of Alkynyl Phosphine Gold(I) Complexes. Angew. Chem. Int. Ed..

[35] Stockland R.A., Kohler M.C., Guzei I.A., Kastner M.E., Bawiec J.A., Labaree D.C., Hochberg R.B. (2006). Organometallic Complexes Containing 17-Ethynyl-17β-hydroxyandrost-4-en-3-one and Related Ethynyl Steroids. Organometallics.

[36] Rana B.K., Nandy A., Bertolasi V., Bielawski C.W., Saha K.D., Dinda J. (2014). Novel Gold(I)- and Gold(III)-*N*-Heterocyclic Carbene Complexes: Synthesis and Evaluation of Their Anticancer Properties. Organometallics.

[37] Rubbiani R., Zehnder T.N., Mari C., Blacque O., Venkatesan K., Gasser G. (2014). Anticancer Profile of a Series of Gold(III) (2-phenyl)pyridine Complexes. ChemMedChem.

[38] Zou T., Lum C.T., Chui S.-Y., Che C.-M. (2013). Gold(III) Complexes Containing *N*-Heterocyclic Carbene Ligands: Thiol “Switch-on” Fluorescent Probes and Anti-Cancer Agents. Angew. Chem. Int. Ed..

[39] Barnard P.J., Berners-Price S.J. (2007). Targeting the mitochondrial cell death pathway with gold compounds. Coord. Chem. Rev..

[40] Holenya P., Can S., Rubbiani R., Alborzinia H., Jünger A., Cheng X., Ott I., Wölfl S. (2014). Detailed analysis of pro-apoptotic signaling and metabolic adaptation triggered by a *N*-heterocyclic carbine-gold(I) complex. Metallomics.

[41] Pratesi A., Gabbiani C., Michelucci E., Ginanneschi M., Papini A.M., Rubbiani R., Ott I., Messori L. (2014). Insights on the mechanism of thioredoxin reductase inhibition by Gold *N*-heterocyclic carbene compounds using the synthetic linear Selenocysteine containing C-terminal peptide hTrxR(488-499): An ESI-MS investigation. J. Inorg. Biochem..

[42] Cheng X., Can P., Alborzinia H., Rubbiani R., Ott I., Wölfl S. (2014). A TrxR inhibiting gold(I) NHC complex induces apoptosis through ASK1-p38-MAPK signaling in pancreatic cancer cells. Mol. Cancer.

[43] Gaspar A., Matos M.J., Garrido J., Uriarte E., Borges F. (2014). Chromone: A Valid Scaffold in Medicinal Chemistry. Chem. Rev..

[44] Keri R.S., Budagumpi S., Pai R.K., Balakrishna R.G. (2014). Chromones as a privileged scaffold in drug discovery: A review. Eur. J. Med. Chem..

[45] Kurzwernhart A., Kandioller W., Bartel C., Bächler S., Trondl R., Mühlgassner G., Jakupiec M.A., Arion V.B., Marko D., Keppler B.K. (2012). Targeting the DNA-topoisomerase complex in a double-strike approach with a topoisomerase inhibiting moiety and covalent DNA binder. Chem. Commun..

[46] Kurzwernhart A., Kandioller W., Bächler S., Bartel C., Martic S., Buczkowska M., Mühlgassner G., Jakupiec M.A., Kraatz H.-B., Bednarski P.J. (2012). Structure–Activity Relationships of Targeted Ru^II^(η^6^-p-Cymene) Anticancer Complexes with Flavonol-Derived Ligands. J. Med. Chem..

[47] Monserrat J.-P., Tiwari K.N., Quentin L., Pigeon P., Jaouen G., Vessières A., Chabot G.G., Hillard E.A. (2013). Ferrocenyl flavonoid-induced morphological modifications of endothelial cells and cytotoxicity against B16 murine melanoma cells. J. Organomet. Chem..

[48] Monserrat J.-P., Al-Safi R.I., Tiwari K.N., Quentin L., Chabot G.G., Vessières A., Jaouen G., Neamati N., Hillard E.A. (2011). Ferrocenyl chalcone difluoridoborates inhibit HIV-1 integrase and display low activity towards cancer and endothelial cells. Bioorg. Med. Chem. Lett..

[49] Kowalski K., Koceva-Chyła A., Szczupak Ł., Hikisz P., Bernasińska J., Rajnisz A., Solecka J., Therrien B. (2013). Ferrocenylvinyl-flavones: Synthesis, structure, anticancer and antibacterial activity studies. J. Organomet. Chem..

[50] Kowalski K., Hikisz P., Szczupak Ł., Therrien B., Koceva-Chyła A. (2014). Ferrocenyl and dicobalt hexacarbonyl chromones—New organometallics inducing oxidative stress and arresting human cancer cells in G2/M phase. Eur. J. Med. Chem..

[51] Kowalski K., Szczupak Ł., Oehninger L., Ott I., Hikisz P., Koceva-Chyła A., Therrien B. (2014). Ferrocenyl derivatives of pterocarpene and coumestan: Synthesis, structure and anticancer activity studies. J. Organomet. Chem..

[52] Kowalski K., Szczupak Ł., Bernaś T., Czerwieniec R. (2015). Luminescent rhenium(I)chromone bioconjugate: Synthesis, photophysical properties, and confocal luminescence microscopy investigation. J. Organomet. Chem..

[53] Patonay T., Pazurik I., Ábrahám A. (2013). C-Alkynylation of Chromones by Sonogashira Reaction. Aust. J. Chem..

[54] Pomestchenko I.E., Polyansky D.E., Castellano F.N. (2005). Influence of a Gold(I)-Acetylide Subunit on the Photophysics of Re(Phen)(CO)_3_Cl. Inorg. Chem..

[55] Sakamoto Y., Moriuchi T., Hirao T. (2015). Organogold(I)-uracil conjugates: Synthesis and structural characterization. J. Organomet. Chem..

[56] Collins A.R. (2004). The comet assay for DNA damage and repair. Mol. Biotechnol..

[57] Ormerod M.G., Collins K.L., Rodriguez-Tarduchy G., Robertson D. (1992). Apoptosis in interleukin-3-dependent haemopoietic cells: Quantification by two flow cytometric methods. J. Immunol. Methods.

[58] Darzynkiewicz Z., Bruno S., Del Bino G., Gorczyca W., Hotz M.A., Lassota P., Traganos F. (1992). Features of apoptotic cells measured by flow cytometry. Cytometry.

[59] Hotz M.A., Gong J., Traganos F., Darzynkiewicz Z. (1994). Flow cytometric detection of apoptosis: Comparison of the assays of *in situ* DNA degradation and chromatin changes. Cytometry.

[60] Koceva-Chyła A., Jędrzejczak M., Skierski J., Kania K., Jóźwiak Z. (2005). Mechanisms of induction of apoptosis by anthraquinone anticancer drugs aclarubicin and mitoxantrone in comparison with doxorubicin: Relation to drug cytotoxicity and caspase-3 activation. Apoptosis.

[61] Kruidering M., Evan G.I. (2000). Caspase-8 in Apoptosis: The Beginning of “The End”?. IUBMB Life.

[62] Alenzi F.Q., Lotfy M., Wyse R. (2010). Swords of Cell Death: Caspase Activation and Regulation. Asian Pac. J. Cancer Prev..

[63] Juo P., Kuo C.J., Yuan J., Blenis J. (1998). Essential requirement for caspase-8/FLICE in the initiation of the Fas-induced apoptotic cascade. Curr. Biol..

[64] McIlwain D.R., Berger T., Mak T.W. (2013). Caspase Functions in Cell Death and Disease. Cold Spring Harb. Perspect. Biol..

[65] Wolf B.B., Schuler M., Echeverri F., Green D.R. (1999). Caspase-3 Is the Primary Activator of Apoptotic DNA Fragmentation via DNA Fragmentation Factor-45/Inhibitor of Caspase-activated DNase Inactivation. J. Biol. Chem..

[66] Sheldrick G.M. (2008). A short history of SHELX. Acta Cryst. A..

[67] Farrugia L.J. (1997). ORTEP-3 for Windows—A version of ORTEP-III with a Graphical User Interface (GUI). J. Appl. Cryst..

[68] Bruno I.J., Cole J.C., Edgington P.R., Kessler M., Macrae C.F., McCabe P., Pearson J., Taylor R. (2002). New software for searching the Cambridge Structural Database and visualizing crystal structures. Acta Cryst. B.

[69] Rubbiani R., Can S., Kitanovic I., Alborzinia H., Stefanopoulou M., Kokoschka M., Mönchgesang S., Sheldrick W.S., Wölfl S., Ott I. (2011). Comparative *in Vitro* Evaluation of *N*-Heterocyclic Carbene Gold(I) Complexes of the Benzimidazolylidene Type. J. Med. Chem..

[70] (2009). Approved Standard-Eighth Edition. M07-A8Method for Dilution Antimicrobial Susceptibility Tests for Bacteria That Grow Aerobically.

[71] Knopik-Skrocka A., Bielawski J. (2005). Differences in amphotericin-B-induced hemolysis between human erythrocytes from male and female donors. Biol. Lett..

