# Supplementary material for: Anticancer and Antibacterial Activity Studies of Gold(I)-Alkynyl Chromones
Source: Molecules. 2015 Oct 30;20(11):19699–718. doi: 10.3390/molecules201119647 (PMC6331995; doi:10.3390/molecules201119647)
Supplement: Supplementary file 1 [file molecules-20-19647-s001.pdf]

molecules

Supplementary Informations

1. DNA Comet Assay

Single cells were embedded in an agarose gel, then treated with lysis buffer, subjected to electrophoresis, stained with a DAPI (4′,6-diamidino-2-phenylindole) solution and analyzed under a fluorescent microscope. Image of a single cell resembles the shape of a comet in which the circular head and tail can be distinguished. The head of the comet consists of a undamaged high molecular weight DNA which in the conditions used does not migrate during the electrophoresis into agarose gel. Damaged DNA of low molecular weight migrates in the tail of the comet in the direction of the anode [58].

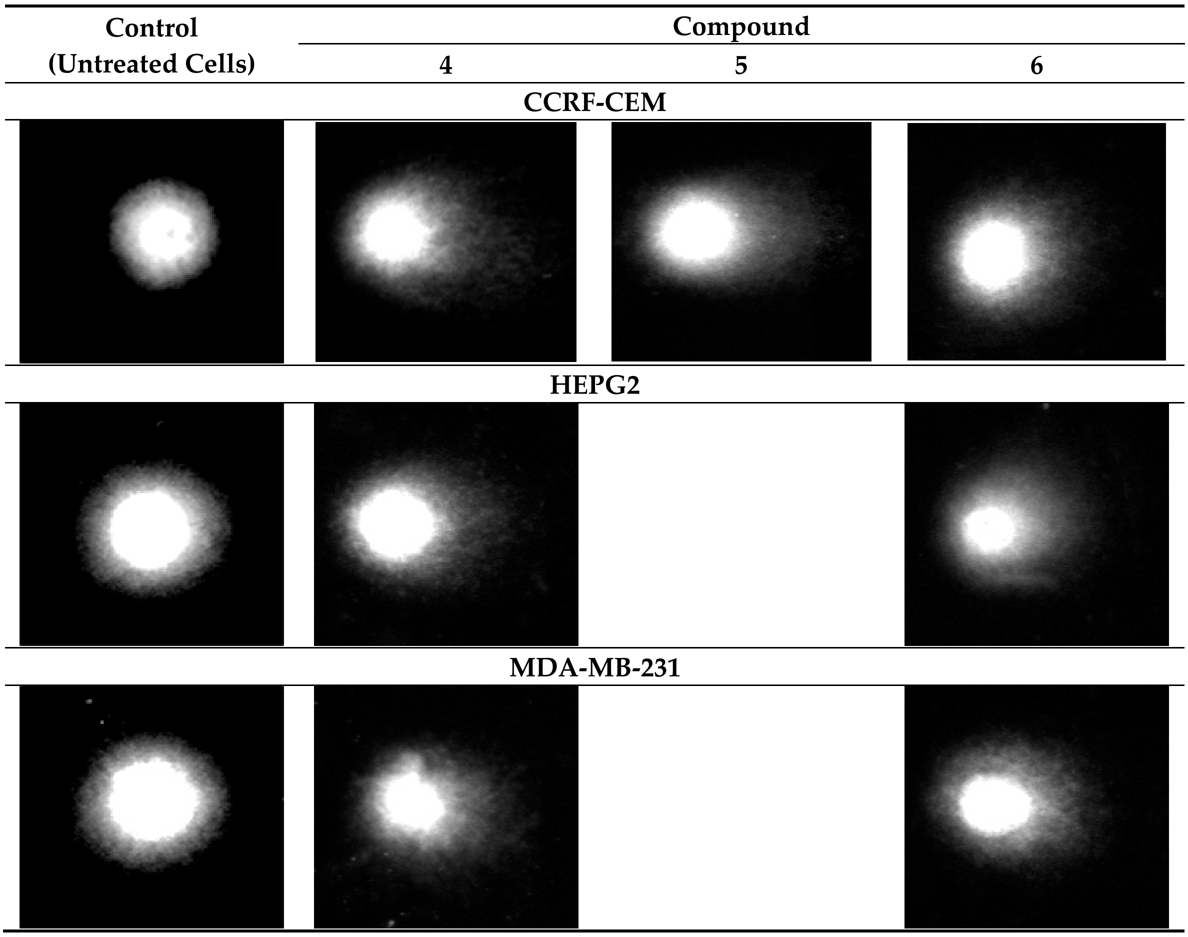

Figure S1. DNA comets obtained from human cancer cells treated for 24 h with complexes **4**, **5** and **6** (single-cell electrophoresis). Pictures taken under a fluorescence microscope (Nikon Eclipse, Tokyo, Japan).

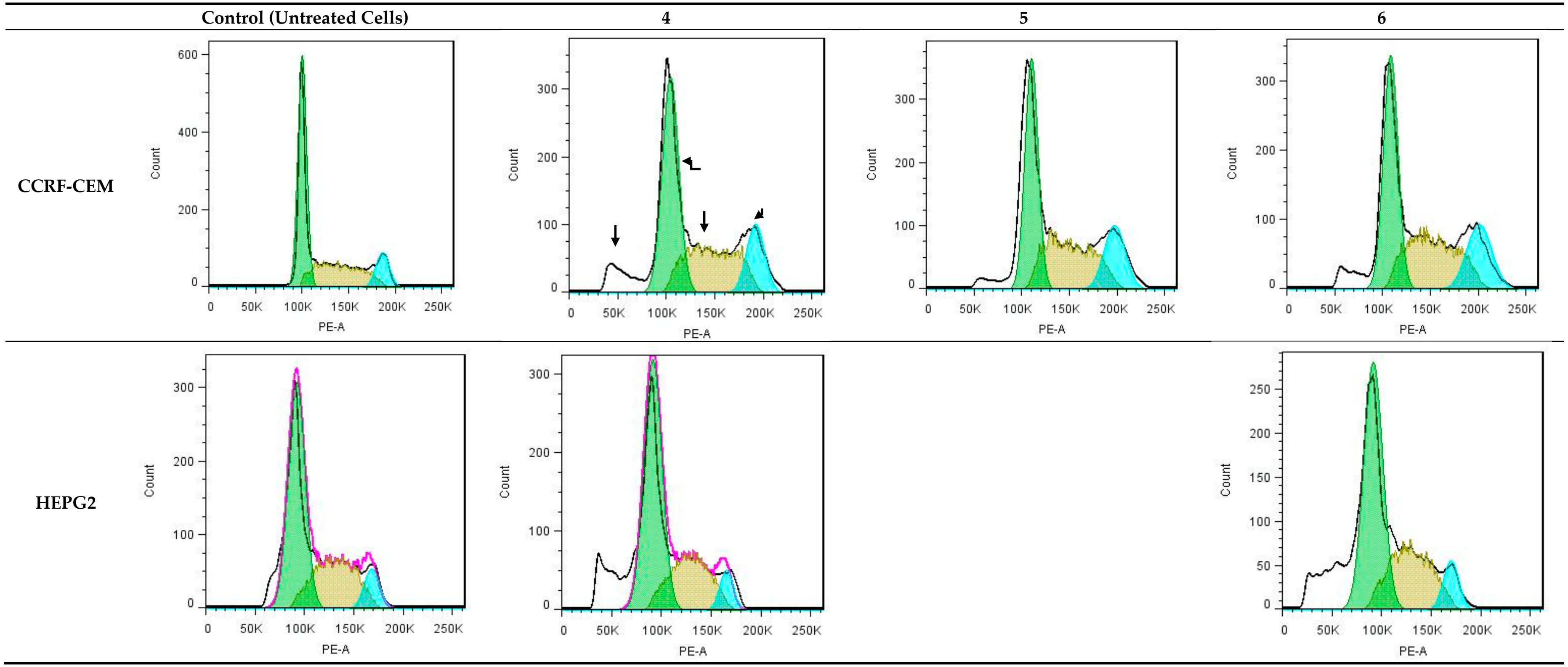

Figure S2. *Cont.*

	**Control (Untreated Cells)**	**4**	**5**	**6**
**MCF-7**	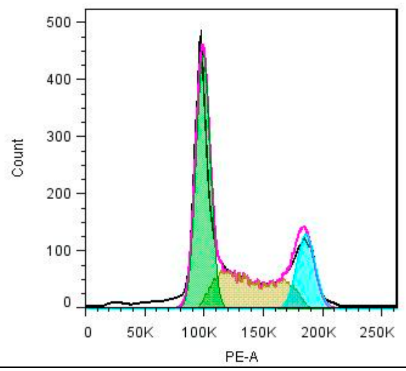	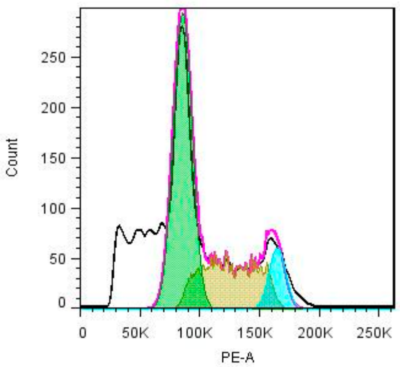	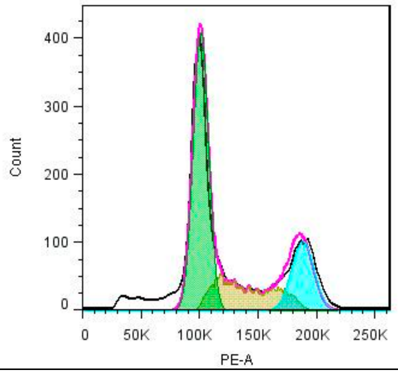	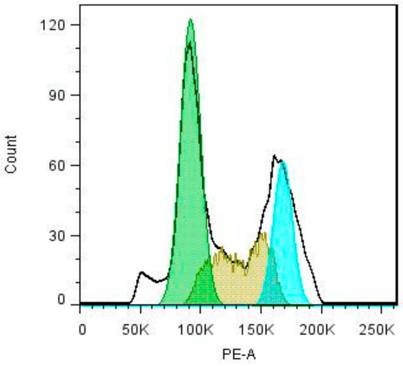
**MDA-MB-231**	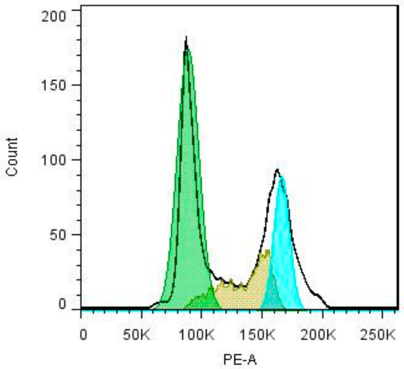	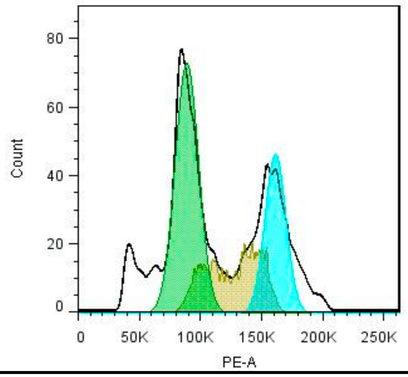		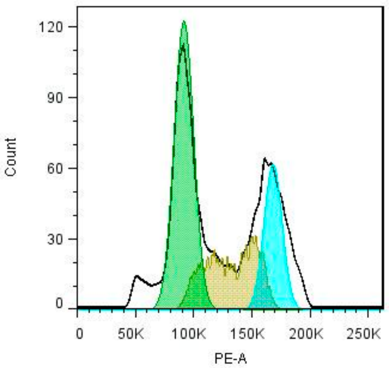

Figure S2. DNA histograms obtained by flow cytometry assay of leukemic (CCRF-CEM) and solid cancer cells (HepG2, MCF-7 and MDA-MB-231) treated for 24 h with complexes **4**, **5** and **6**.

Table S1. Crystallographic and structure refinement parameters for **5**·CH_2Cl_2·0.5 C_4H_{10} and **6**.

	**5·CH_2Cl_2·0.5 C_4H_{10}**	**6**
Chemical formula	$C_{48}H_{46}AuCl_2FeNO_3P$	$C_{35}H_{24}AuO_2P$
Formula weight	1039.54	704.48
Crystal system	Monoclinic	Monoclinic
Space group	*P* 2_1/*c* (no. 14)	*P* 2_1/*c* (no. 14)
Crystal color and shape	Orange block	Colorless block
Crystal size	0.25 × 0.22 × 0.21	0.20 × 0.19 × 0.17
a (Å)	10.8484(6)	14.0444(6)
b (Å)	15.1395(5)	12.6413(5)
c (Å)	25.8631(12)	32.0520(12)
β (°)	90.475(4)	101.487(3)
V (Å^3)	4247.6(3)	5576.5(4)
Z	4	8
T (K)	173(2)	173(2)
D_c (g·cm^{-3})	1.626	1.678
μ (mm^{-1})	3.998	5.364
Scan range (°)	1.56 < θ < 29.23	1.48 < θ < 29.26
Unique reflections	11509	15096
Observed refls [I > 2σ(I)]	7473	8310
R_{int}	0.0839	0.0905
Final *R* indices [I > 2σ(I)] *	0.0358, wR_2 0.0706	0.0300, wR_2 0.0367
R indices (all data)	0.0715, wR_2 0.0766	0.0841, wR_2 0.0423
Goodness-of-fit	0.837	0.703
Max, Min Δϱ/e (Å^{-3})	1.550, −0.942	1.103, −1.104

* Structures were refined on F_0^2: $wR_2 = [\Sigma[w (F_0^2 - F_c^2)^2]/\Sigma w (F_0^2)^2]^{1/2}$, where $w^{-1} = [\Sigma(F_0^2) + (aP)^2 + bP]$ and $P = [\max(F_0^2, 0) + 2F_c^2]/3$.
